# Generation of a Nanobody Targeting the Paraflagellar Rod Protein of Trypanosomes

**DOI:** 10.1371/journal.pone.0115893

**Published:** 2014-12-31

**Authors:** Emmanuel Obishakin, Benoit Stijlemans, Julien Santi-Rocca, Isabel Vandenberghe, Bart Devreese, Serge Muldermans, Philippe Bastin, Stefan Magez

**Affiliations:** 1 Cellular and Molecular Immunology, Vrije Universiteit Brussel, Pleinlaan 2, B-1050 Brussels, Belgium; 2 Structural Biology Research Center, VIB (Flanders Institute for Biotechnology), Pleinlaan 2, B-1050 Brussels, Belgium; 3 Laboratory of Myeloid Cell Immunology, VIB, (Flanders Institute for Biotechnology) Pleinlaan 2, B-1050 Brussels, Belgium; 4 Laboratory for Protein Biochemistry and Biomolecular Engineering (L-ProBE), Department of Biochemistry and Microbiology, Ghent University, K.L Ledeganckstraat 35, 9000, Ghent, Belgium; 5 Trypanosome Cell Biology Unit, Institut Pasteur & CNRS, URA 2581, 25 rue du Docteur Roux, 75015 Paris, France; London School of Hygiene and Tropical Medicine, United Kingdom

## Abstract

Trypanosomes are protozoan parasites that cause diseases in humans and livestock for which no vaccines are available. Disease eradication requires sensitive diagnostic tools and efficient treatment strategies. Immunodiagnostics based on antigen detection are preferable to antibody detection because the latter cannot differentiate between active infection and cure. Classical monoclonal antibodies are inaccessible to cryptic epitopes (based on their size-150 kDa), costly to produce and require cold chain maintenance, a condition that is difficult to achieve in trypanosomiasis endemic regions, which are mostly rural. Nanobodies are recombinant, heat-stable, small-sized (15 kDa), antigen-specific, single-domain, variable fragments derived from heavy chain-only antibodies in camelids. Because of numerous advantages over classical antibodies, we investigated the use of nanobodies for the targeting of trypanosome-specific antigens and diagnostic potential. An alpaca was immunized using lysates of *Trypanosoma evansi*. Using phage display and bio-panning techniques, a cross-reactive nanobody (Nb392) targeting all trypanosome species and isolates tested was selected. Imunoblotting, immunofluorescence microscopy, immunoprecipitation and mass spectrometry assays were combined to identify the target recognized. Nb392 targets paraflagellar rod protein (PFR1) of *T. evansi*, *T. brucei*, *T. congolense* and *T. vivax*. Two different RNAi mutants with defective PFR assembly (*PFR2^RNAi^* and *KIF9B^RNAi^*) were used to confirm its specificity. In conclusion, using a complex protein mixture for alpaca immunization, we generated a highly specific nanobody (Nb392) that targets a conserved trypanosome protein, i.e., PFR1 in the flagella of trypanosomes. Nb392 is an excellent marker for the PFR and can be useful in the diagnosis of trypanosomiasis. In addition, as demonstrated, Nb392 can be a useful research or PFR protein isolation tool.

## Introduction

Trypanosomes are a family of haemoflagellates that cause chronic infections in humans and livestock. Human African Trypanosomiasis (HAT) is caused by *Trypanosoma brucei gambiense* and *Trypanosoma brucei rhodesiense* [1]. Animal African Trypanosomiasis (AAT) known as nagana is mainly caused by *Trypanosoma vivax, Trypanosoma congolense* and *Trypanosoma brucei brucei* [2], [3]. *Trypanosoma evansi* and *Trypanosoma equiperdum* cause forms of AAT referred to as surra and dourine respectively [4], [5]. In animals, trypanosomiasis is usually characterised by undulating fever and parasiteamia, progressive anaemia, loss of condition, abortions and immunodeficiencies [6], [7].

As there is no effective vaccine against trypanosomiasis [8], treatment of HAT and AAT is limited to a few drugs that in turn generate serious side effects and have recently been facing drug resistance [9]. Early detection and control is the only way to prevent outbreaks. Hence, there is a need for sensitive and specific diagnostic measures [10], [11].

Parasitological and serological techniques are currently used for the diagnosis of trypanosome infections but are limited by their low sensitivity. Antibody detecting serological tests such as indirect-ELISA, indirect immunofluorescence tests and card agglutination are also used, however, they lead to largely presumptive diagnosis because active infections are not verifiable and distinctions between cured and uncured cases cannot be made. In addition, specificity and sensitivity of these tests require further evaluation. Recently, research has turned towards methods for antigen detection [12], [13].

In addition to the conventional IgG antibody, the immune system of camelids produce heavy-chain antibodies (HCAbs) which are devoid of light chains, lack CH1 domains in their heavy chain and are capable of antigen recognition [14]. In camels, 50–80% of immunoglobulins are heavy chain-only antibodies while in South American camelids (llama, alpaca, guanaco and vicugna) about 10–25% belong to this group [15]. The antigen binding part of the single domain antibody can be produced by recombinant expression in bacteria commonly referred to as Nanobody (Nb). With a dimension of ∼4×2.2 nm, a molecular weight of 15 kDa and high affinity and specificity for their targets, they have the ability to specifically recognize cryptic epitopes that are not easily accessed by classical antibodies [15], [16]. In addition, they are resistant to chemical and thermal denaturation and their low production cost makes them attractive. They are now being used as crystallization chaperones in solving protein structures [17]. In addition, nanobody technology has been recently introduced as tools in malaria and cancer research [18], [19]. Recently, the use of nanobodies in trypanosome research has been explored [16], [20], [21].

Compared to monoclonal antibodies in research and immunodiagnostics, nanobody use is relatively new and promising. Although some nanobodies that recognize trypanosome antigens have been generated [5], isolation of target protein antigens out of complex sample mixtures using Nbs has not been shown. Here, we demonstrate the development of Nb targeting specifically a conserved protein among various trypanosome species and subsequent isolation and identification of the target protein out of total trypanosome lysates. We also present one-step direct nanobody based immunofluorescence labelling for diagnosis of AAT.

## Material and Method

### Ethics statement

Approval for animal experiments was obtained from the Animal Ethical Committee of the Vrije Universiteit Brussel (Ethics committee protocol number 10-220-5).

### Trypanosome antigen preparation, alpaca immunisation and lymphocyte isolation

Total parasite lysates were prepared as described before [5] (**S1 Material and Methods**). 100 µg each of five different lysates (*T. evansi* STIB 816, *T. evansi* ITMAS *0101399B, T. evansi itmas 150399B*, *T. evansi* ITMAS *120399C* and *T. evansi itmas 150399C*) were pooled and used to immunize an alpaca with six subcutaneous injections at weekly intervals. 500 µl of lysate was added to 500 µl of Gerbu adjuvant LQ 3000 (GERBU Biotechnik) per injection [17]. Four days after the last boost, 75 ml of anti-coagulated blood was collected from the alpaca and lymphocytes were isolated using Lymphoprep (Nycomed) according to the manufacturer's instruction. The immunization and blood collection was done by Alpa-Vet (www.alpa-vet.be). Lymphocytes were separated by gradient centrifugation for 25 minutes at 2,000 rpm using an Eppendorf 5810 centrifuge at room temperature. 5×10^7^ lymphocytes were lysed by adding 1 ml of Trizol in Eppendorf tubes.

### Construction of anti-trypanosome Nb library and selection of specific antibody fragments (Biopanning)

Library construction was done as described [5], [22]. Briefly, mRNA extracted from the Trizol pellet was used as a template for RT-PCR to generate a first strand cDNA using oligo-dT primers. cDNA was used as template to amplify 2 different products using specific primers CALL001 and CALL002 [22] which yielded the *VH* and the *VHH* containing gene fragments of 900 and 600 bp, respectively. The 600 bp product was excised from 1% agarose gel, used as template for a second PCR via nested primers A4short and 38 [5] to amplify the *VHH* sequence of about 400 bp and restricted with Not I and PstI restriction enzymes (Roche). The PCR fragments were ligated into the phagemid vector pHEN4 [23] and transformed into electro-competent *E.coli* TG1 cells. The Resulting nanobody library was super-infected with M13K07 helper phages for the expression of nanobodies on the phages. Biopanning was performed as described [22] by coating ELISA plates (Nunc) with 1 µg amount of *T. evansi* STIB per well. Phage enrichment was achieved by 3 rounds of *in vitro* selection. Phages were eluted as described [22]. TG1 *E. coli* cells were infected with the eluted phages, and selected from LB-ampicillin plates. One hundred viable colonies were randomly picked from each round of panning and their *VHH* was expressed as described [22]. The periplasmic extracts (PE) were obtained through osmotic shock as described [24]. The enrichment of each round of panning was checked by a polyclonal phage ELISA with anti-M13-HRP antibodies [5]. Each colony PE from round two and three was tested on PE-ELISA using mouse anti-heamaglutinin antibodies [5] and further tested on two other lysates (*T. evansi* 0101399B, *T. evansi* 150399B) for cross reactivity.

### Expression and purification of antibody fragments for ELISA testing

The cloned insert that expressed protein recognizing various trypanosome lysates, was sequenced using the RP or GIII primer [17] and sequences were grouped based on the differences in their complementarity determining regions (CDRs). Representatives of each group were recloned into the expression vector pHEN6 using Eco91I (Thermo scientific) and Pst I (Roche) enzymes. The plasmid constructs were transformed into WK6 *E. coli* cells and large quantities of His_6_ tagged recombinant Nb were expressed as described [22] following periplasmic expression through osmotic shock as described [24]. The PE was initially purified using 1 ml of HIS-Select Nickel Affinity Gel (Sigma-Aldrich) and elution was performed with 1 column volume of 0.5 M imidazole repeated 3 times. The elution was purified on Hiload Superdex-75 (16/600) gel filtration column (Aktaxpress, GE Healthcare) as described [22]. Solid phase ELISA was performed by coating of different trypanosome lysates (5 µg/well) on Maxisorb 96 well plates (Nunc) at 4°C overnight. The ELISA was performed as described [5] using purified Nb as primary antibody, followed by in-house generated rabbit polyclonal anti-VHH IgG and a goat anti-rabbit-IgG antibody conjugated to horseradish peroxidase (Sigma-Aldrich). A non-relevant nanobody (NbBCII10) [22] was used as negative control for antibody detection while a non-relevant protein, Bovine serum albumin (BSA) was used as negative control for lysate coatings.

### Flow cytometry and Immunofluorescence Assay (IFA)

From the result of ELISA using purified nanobodies, Nb392 was selected and labeled directly by conjugation with ALEXA Fluor 488 (Molecular Probes) according to the manufacturer's instructions. The labeled Nb392 was used for flow cytometry and immunofluorescence assays on purified, fixed and permeabilised (**S1 Material and Methods**) bloodstream forms of *T. evansi* STIB 816, *T. brucei brucei* AnTat1.1, *T. congolense* Tc13 and *T. vivax* IL700 strains. ALEXA labelled NbBCII10 was used as negative control.

### Western blotting

To identify the target of Nb392 in the flagella, western blots were performed on total lysates as described [16] using Nb392 probed with in-house generated rabbit polyclonal anti-VHH IgG and a goat anti-rabbit-IgG antibody conjugated to horseradish peroxidase (Sigma-Aldrich). For phenotypic confirmation that Nb392 targets PFR1/2, IFA and immunoblotting were conducted on RNAi mutants of PFR protein, *PFR2^RNAi^* [25] and *KIF9B^RNAi^* [26] mutant (procyclic) parasites using same protocols as performed on parasites and lysates above (**S1 Material and Methods**) Anti-ALBA antibodies were used as loading controls [27].

### Antigen identification by immunoprecipitation of flagella extracts and mass spectrometry

Flagella extraction was performed on T. evansi STIB816 parasites using Triton X-100 as described [28] (S1 Material and Methods, S1 Fig.). Nb392 was immobilized covalently on N-hydroxysuccinimide (NHS) activated dry agarose resin according to the manufacturer's instructions (Thermo scientific pierce) overnight at 4°C. After quenching the unbound sites with 1.0 M Tris-HCl pH 7.4, flagella extracts was added at room temperature for 3 hours, washed with PBS, acidic elution was performed with 0.2 M glycine-HCl, pH 2.5 according to the manufacturer's instructions. 1–3 µg of the eluted protein was resolved by SDS-PAGE using a 12.5% premade polyacrylamide gel, later stained with 0.1% commassie Brilliant Blue R-250 in 40% Methanol for 1–2 minutes, followed by washing with 50% methanol. The resulting two bands were excised separately for mass spectrometry.

### Maldi-MS of gel bands

The protein bands were exiced from gel and digested with trypsin in ammoniumbicarbonate. Digestion and peptide extraction were performed according to standard protocols [29] (**S1 Material and Methods**). Sample Ms and tandem Ms spectra were acquired on a 4800 Proteomics Analyzer. Data analyses were performed using 4000 Series Explorer and Data explorer software. Protein identification was obtained by applying the Mascot Search Engine against the Swissprot database, taxonomy eukaryotes and implying decoy database searches. The parameters were set to mono-isotopic mass values using peptide charge +1 and the peptide mass tolerance was set to 200 ppm, with a maximum of missed cleavages of two. Methionine oxidation was set as a Variable modification; the significance tolerance threshold was set below 0.05. Protein identification was derived from peptide fragmentation spectra using the fragmentation ion simulator of the Data explorer software.

### Statistical Analysis

FACS data were analysed using Flowjo software and ELISA data were translated into graphical representations using Prism software (GraphPad Prismv.4.0, GraphPad Software Inc. San Diego, CA). ELISA experiments were performed minimum of three times with lysates coated in triplicates. Data are presented as mean values ± SD. Student's t test was used to compare means. If p > 0.05, differences were considered significant. Asterisks were used to indicate the degrees of significance (*P<0.05, **P<0.01, ***P<0.001).

## Results

### Anti-trypanosome Nb library construction and selection of specific nanobody fragments

**Figure 1:**
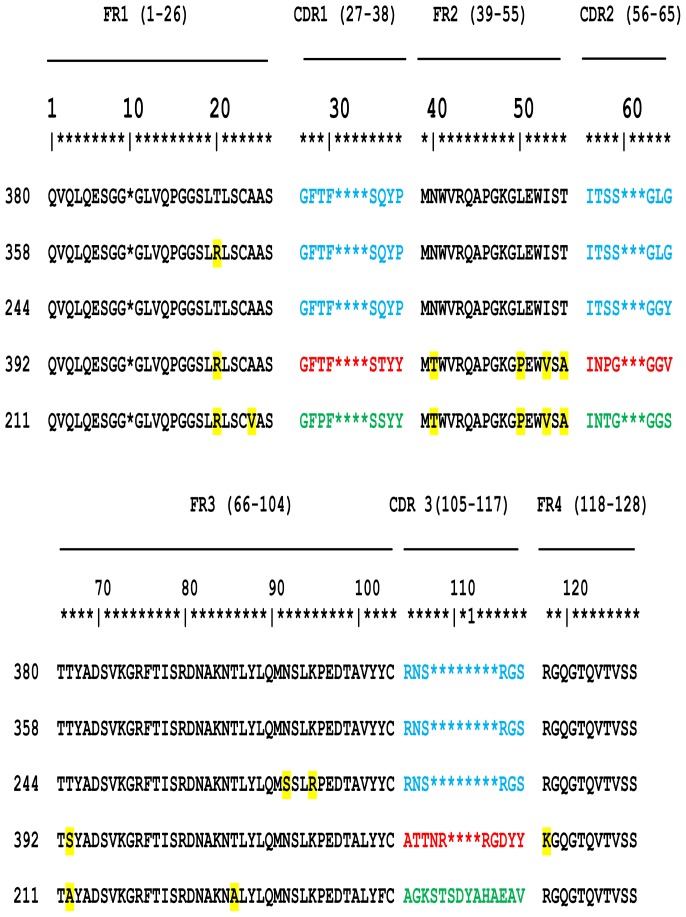
Sequence alignment of deduced amino acid. Sequences of the different cross-reactive nanobodies highlighting the framework (FR) and complementarity determining region (CDR) sequences. CDR sequences are highlighted in blue, red and green (corresponding to different families), while FR differences are marked in yellow. The guidelines of the international Immunogenetics information system of numbering are used (http:/imgt.cines.fr).

Gene fragments of heavy chain antibodies from the immunized alpaca were cloned into the phagemid vector pHEN4 and used to construct a Nb library containing ∼2.26×10^7^ individuals by transforming TG1 *E.coli* cells. Colony PCR showed ∼80% of the clones contained the expected insert size (∼700 bp). Compared binding capacity of phages from three rounds showed the highest ELISA OD readings at the third round, suggesting a progressive enrichment. Furthermore, ELISA tests with PE extracts of individual clones revealed that 39 colonies out of 49 reacted on 2 different *T. evansi* lysate (data not shown). *VHH* genes of these clones were cloned into the pHEN6 vector and sequenced for confirmation. Clones were transformed into WK6 *E. coli* cells for large-scale periplasmic expression. Amino acid sequence analysis of the binders showed five nanobody groups but three distinct families based on CDR3 differences (Fig. 1). A representative of each family (Nb211, Nb358, and Nb392) was expressed and purified. Out of the 3 selected Nbs, Nb392 showed the highest OD value in an ELISA on 5 strains of *T. evansi* tested (data not shown) and therefore was selected for further analysis.

### Nb392 detects an intraflagellar antigen

**Figure 2:**
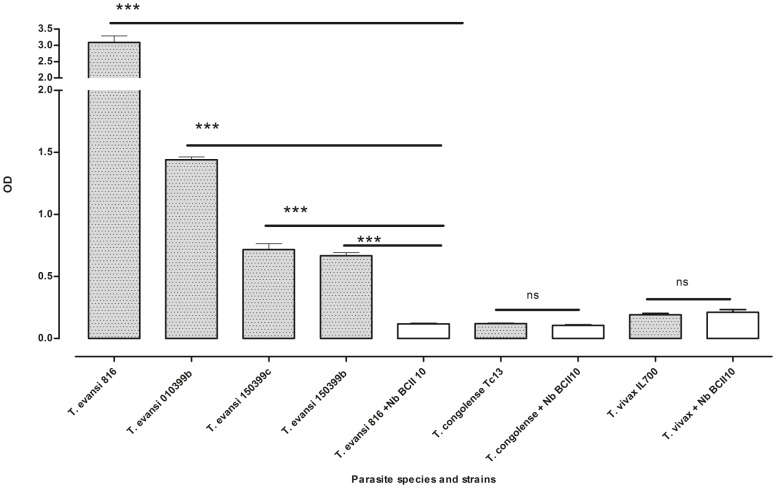
Solid-phase ELISA result. Coating 5 µg/well of soluble protein of parasite lysates (*T. evansi* strains, *T. congolense and T. vivax*) and subsequent recognition by Nb392 and non-relevant nanobody (NbBCII10) as negative control. In each of three separate experiments, lysates were plated in triplicates and detected with Nb392 (shaded boxes) or NbBCII10 (unshaded boxes). Data presented are mean values of three wells (±SD). The mean values of negative control wells are compared to the mean values of corresponding test wells coated with lysates of *T. evansi* strains, *T. vivax* and *T. congolense*, *P<0.05, **P<0.01, ***P<0.001. Results shown are representative of three independent experiments.

**Figure 3:**
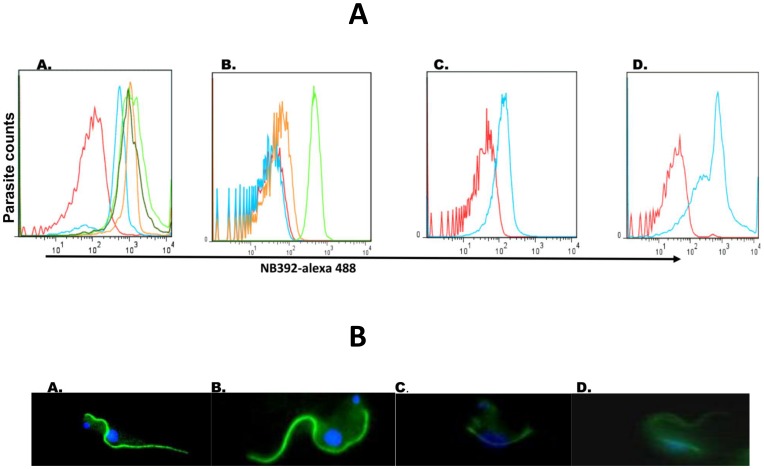
A. Flowcytometric profiles showing binding of ALEXA-labeled Nb392 to fixed and permeabilised trypanosomes. Fixed and permeabilised cells: unstained (red) and stained (blue) *T. evansi* STIB 816, stained *T. evansi Itmas 010399b* (light green), stained *T. evansi Itmas 120399b* (dark green), stained *T. evansi Itmas 150399b* (orange); B) Unstained live *T. brucei* AnTat1.1 (red), fixed and permeabilised *T. brucei* AnTat1.1 unstained (blue), stained (green), stained with control NbBCII 10 (orange); C) Fixed and permeabilised *T. vivax IL700* unstained (red) or stained (blue); D) Fixed and permeabilised *T. congolense* Tc13 unstained (red) or stained (blue). **B. Direct Immunofluorescense assay of fixed and permeabilised trypanosomes.** Fixed and permeabilised trypanosomes incubated with Alexa 488- labeled Nb392 (green) and with DAPI (blue) staining nuclear and mitochondrial DNA. A) *T. evansi* STIB 816; B) T. *brucei; C) T. congolense Tc13; D) T. vivax IL700.* Magnification; X1000.

In ELISA, BSA protein was coated on ELISA plate as negative control antigen. Using Nb392 for detection, the mean value of OD of the BSA coated wells is 0.083 +/− 0.012, which is considered to be equal background level (data not shown). A non-relevant nanobody (NbBCII10) was used as a negative control antibody for all the parasite species as well. When compared, the results show that the signals obtained using Nb392 on *T. evansi* STIB816, *T. evansi itmas 0101399B, T. evansi itmas 150399B*, and *T. evansi itmas 150399C* were significant. In contrast, Nb392 signal on *T. congolense* and *T. vivax* coating gave similar results as those obtained with the irrelevant control NbBCII10 (Fig. 2). By flow cytometry, ALEXA-488 labeled Nb392 showed no signals on live cells but bound fixed and permeabilised *T. evansi* parasites. Similarly, fixed and permeabilised *T. brucei*, *T. congolense* and *T. vivax* parasites showed positive signals using labeled Nb392 by flow cytometry (Fig. 3A). These results were confirmed on direct immunofluorescence assay using ALEXA-labeled Nb392. While live parasites were completely negative, fixed and permeabilised *T. evansi*, *T. brucei*, *T. vivax* and *T. congolense* parasites showed bright staining only in the flagellum (Fig. 3B). Results of both flow cytometry and direct IFA indicate that Nb392 target is localized within the flagellum.

### Identification of the Nb392 target protein

To determine the size of the specific protein recognized by Nb392, immunoblotting was initially performed on parasite lysates. Two close but distinct bands of approximately 70 and 73 kDa were consistently detected. Next, immunoprecipitation was performed by capturing the target protein from flagella crude extracts through the covalent immobilization of Nb392 on NHS activated dry agarose resin. Eluted fractions were resolved by SDS-PAGE. Two protein bands similar to what was observed by western blotting appeared on the gel when stained with Commassie blue. The two bands designated 1 (upper) and 2 (lower) were analyzed via MS. The mass spectra of the tryptic peptides (S2A and S2B Figs.) of protein samples all match with tryptic peptides derived of the 73 kDa paraflagellar rod protein 1 (PFR1) protein (Table 1).

**Table 1:** List of peptide masses from mass spectra of upper and lower bands and their respective sequence matches with the positions in the genome of *T. brucei*.

Peptide masses (Da) in upper protein band	Peptide masses (Da) in lower protein band	sequence	position
1316,4698	1316,4698	(R)IEEIDREEK(R)	346(7)–356(7)
1301,1904	1301,1904	AEELVAAVDVGTK	57–69
-	1193,4562	LGTERFDEVK	336–345
1049,3983	1049,3983	ELYRPEDK	93–100
1664,7115	-	VEYSQFLEVASQHK	359–372
-	1707,5337	CTGLVEELVSEGCAAVK	388–404
-	1585,5837	LDVHKEHLEYFR	419–430
-	1566,4888	TSQDLAALRLDVHK	410–423
1876,9585	-	MVEYRSHLTKQEEVK	539–553
1841,9967	-	SQLDATQLAQVPTQTLK	149–165
1349,7171	1349,7171	LGTERFDEVKR	336–346
1260,731	1260,731	LIDLIQDKFR	227–236
1214,6851	1214,6851	IAAEREEIKR	554–563
1141,6436	1141,6436	VLQDLRQNR	112–120

### Confirmation of Nb392 reactivity using trypanosome PFR mutants

**Figure 4:**
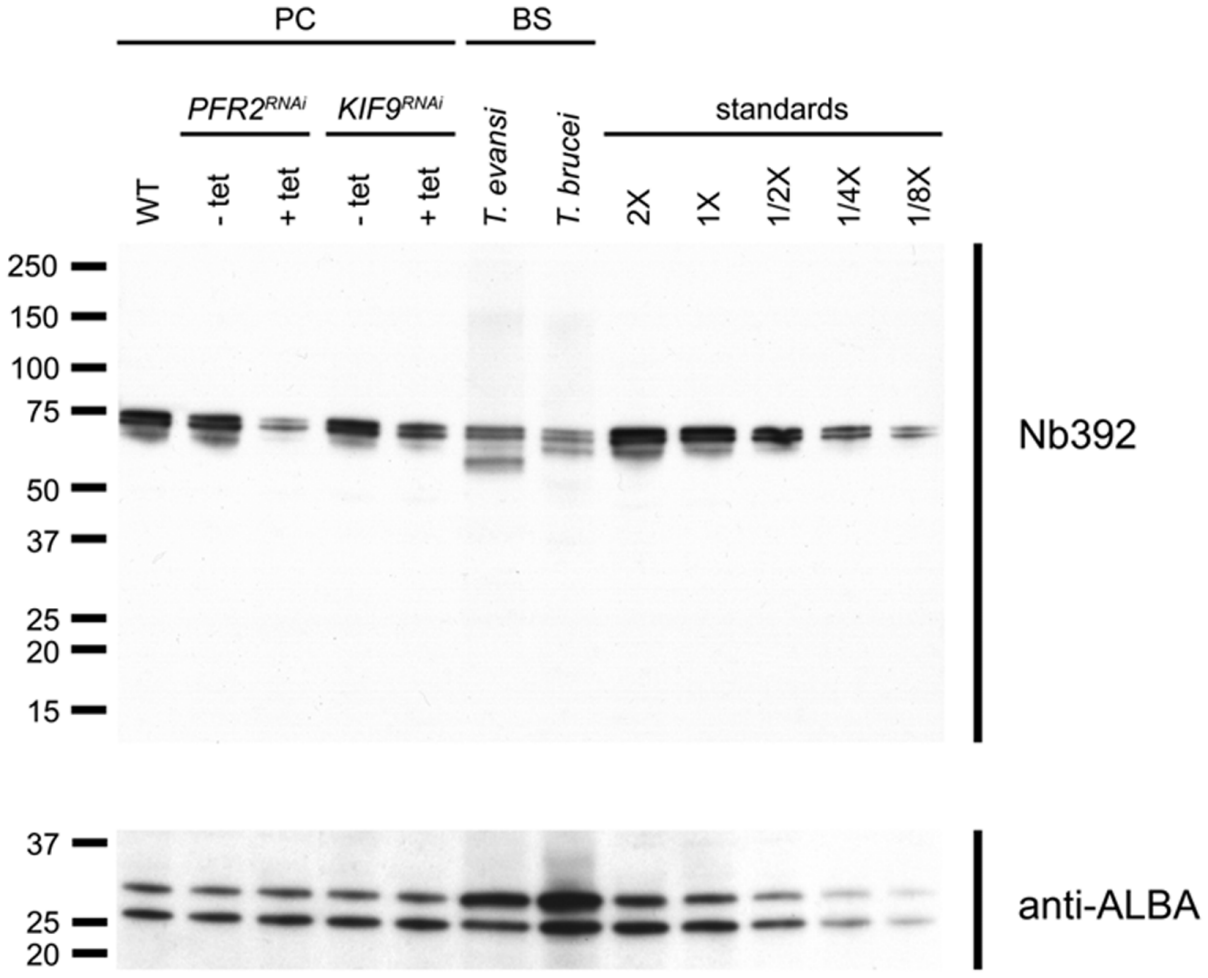
Western blot analysis on wild type and mutant parasites probed with Nb392 and anti-ALBA as loading control. From left to right upper panel: WT *T. brucei* procyclics, *PFR2^RNAi^* uninduced, *PFR2^RNAi^* 2-day induction, *KIF9B^RNAi^* uninduced, *KIF9B^RNAi^* 4-day induction *T. evansi* blood form, *T. brucei* WT blood form, *T. brucei* procyclics WT calibration with 2×10^6^, 1×10^6^, 5×10^5^, 2.5×10^5^ and 1.25×10^5^ parasites. Lower panel: Anti-ALBA antibody used as loading control. Values on the left are given in kilodaltons.

**Figure 5:**
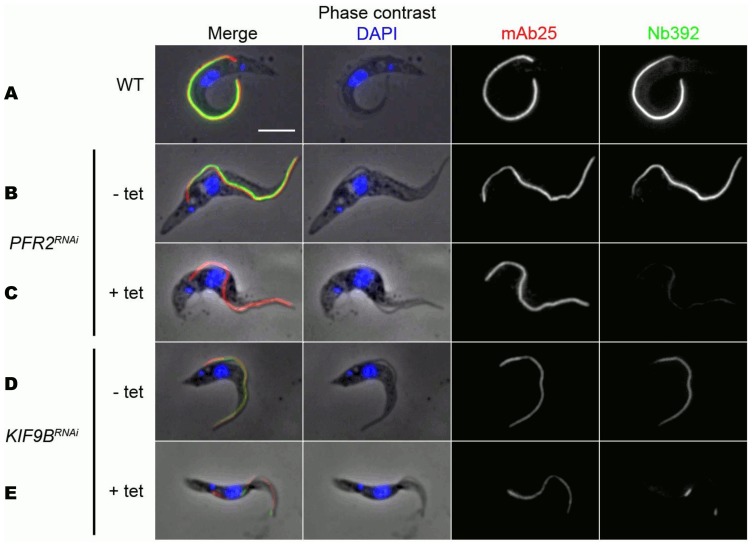
Immunofluorescence assay on *T. brucei* procyclic cells showing reactivity of Nb392 in two PFR mutants. Horizontal rows from top to bottom: Wild type (A) *PFR2^RNAi^* uninduced (B), *PFR2^RNAi^* induced (C), *KIF9B^RNAi^* uninduced (D), *KIF9B^RNAi^* induced (E), Vertical columns from left to right: combined phase-contrast DAPI, mAb25 and Nb392 images (left), DAPI images (middle left), mAb25 (middle right), Nb392 (right). Scale bar: 5 µm.

The *PFR2^RNAi^ T. brucei* procyclic strain expresses double stranded RNA of the *PFR2* gene under the control of a tetracycline inducible promoter [25]. When RNAi is activated, PFR2 is not expressed and only a rudimentary PFR structure is assembled that nevertheless still contains PFR1 [25], [30]. Immunoblotting with Nb392 revealed 85% reduction in PFR signal intensity 2 days after RNAi induction (Fig. 4). Double immunofluorescence was performed using Nb392 and the monoclonal antibody mAb25 [31] as an independent axoneme marker. While the axoneme marker exhibited normal flagella staining, Nb392 only showed a very faint staining, indicating the presence of PFR1 (Fig. 5A–C).

KIF9B is a flagellar kinesin essential for correct assembly of the PFR. When absent, trypanosomes assemble flagella of normal length but with defective PFR distribution [26]. To further confirm if Nb392 binds to PFR proteins, immunoblotting was performed using the *KIF9B^RNAi^* mutant [26]. When *KIF9B* was silenced, a 67% reduction in both PFR bands' intensity was detected by imunoblotting using Nb392, as compared to wild type parasites (Fig. 4). Likewise, IFA revealed discontinuous Nb392 signal all along the length of the flagellum (Fig. 5D–E). Collectively, results demonstrate that the antigen detected by Nb392 behaves as expected for PFR1 in two distinct mutant contexts.

## Discussion

In this study, we produced a nanobody termed Nb392 and demonstrated it recognises PFR1. Generated originally against a *T. evansi* sample, Nb932 was shown to also recognize PFR homologues in *T. brucei*, *T. congolense* and *T. vivax*, indicating its potential use for the development of diagnostic tools for AAT. Moreover, a novel nanobody based immunoprecipitation is reported, permitting the isolation of the target protein from a complex mixture of trypanosome soluble extract.

Development of sensitive diagnostic methods readily available on the field remains a priority in trypanosomiasis control [32]. Microscopic examination is the most common diagnostic measure on the field but it lacks sensitivity. Antigenic variation and low antibody turnover in active or chronic infections make antigen detection and capturing methods to be preferable over antibody detection methods. Moreover, a test of cure cannot be performed as antibodies that recognize diagnostic antigen can remain in the serum after treatment [12], [33]. However, classic mouse monoclonal antibodies face biological (inaccessibility to cryptic epitopes) and practical (high cost of production, necessity for a cold chain incompatible with conditions encountered in the field) issues, prompting a search for antibodies of high affinity, high thermo-stability, access to cryptic epitopes and low production costs. To improve detection and antigen capturing in trypanosome research, we investigated the alternative use of nanobodies.

Antibody selection for antigen capturing is usually done by immunizing an animal with a known ‘preferred’ protein target [17] or with complex protein mixtures such as total lysates [34]. So far, using specific proteins has not been successful in developing antigen-specific tests for trypanosomiasis. We therefore, preferred an inverse proteomics approach. Immunizing with complex protein mixtures is beneficial: first, antibodies are produced against mixed antigen as in natural infection. Second, purified or recombinant antigen is not essential for immunization and screening. Third, antibodies against post-translational modified variants can be elicited. Fourth, selection of an antibody recognizing a dominant antigen in a properly folded native form is favoured.

Here, an alpaca was immunized with whole soluble extract of *T. evansi* STIB816 parasites. Nanobody gene fragments were selected by panning the generated VHH library on the same antigen as bait and tested for cross-reactivity to other trypanosome species and strains. In flow cytometry analysis and IFA, Nb392 was shown to be cross-reactive to all *T. evansi* tested and to *T. brucei*, *T. vivax* and *T. congolense* species, exclusively binding to the flagellum on IFA. In solid phase ELISA, Nb392 binds total lysates of *T. evansi* STIB 816 and all other *T. evansi* strains while also recognizing *T. brucei AnTat* 1.1 strain. This can be attributed to close relatedness of *T. evansi* and *T. brucei* species [35]. The signals obtained by using Nb392 on *T. evansi strains* were significantly different from the signals obtained using an irrelevant nanobody (NbBCII10) while signals using Nb392 on *T. congolense* and *T. vivax* protein extracts were not significantly different from the signals obtained by using the irrelevant nanobody on the same lysates, hence considered negative. This indicates that the target is less conserved in these species when compared to *T. evansi* and *T. brucei* species. Furthermore, ELISA was performed using total lysates, this probably resulted in competitions between the target protein and numerous other proteins while IFA was performed on intact, fixed and permeabilised parasites, this may have contributed to the weak signals observed in ELISA using *T. vivax* and *T. congolense*. Moreover, IFA and flow cytometry could both be more visually sensitive than ELISA.

Considering the IFA flagella signals and imunoblotting showing that Nb392 recognizes two trypanosome flagella proteins between 70–73 kDa, Nb392 was used for immunoprecipitation of flagella proteins. Mass spectra of all the tryptic peptides of the two protein bands captured by Nb392 matched with tryptic peptides of PFR1 proteins, confirming that in natural conditions used for immunoprecipitation and IFA, Nb392 binds to PFR1. The lower band could result from subtle proteolytic cleavage of PFR1 or due to sensitivity of PFR proteins to oxidation or reduction, shifting their migrating pattern as previously observed [36].

To confirm that Nb392 targets PFR, we used both *PFR2^RNAi^* and *KIF9B^RNAi^* mutant parasites. In *PFR2^RNAi^* mutants, Nb392 showed a much-reduced signal, in agreement with the presence of a rudimentary PFR structure composed of PFR1 [30], hence indicating PFR1 as a primary target of Nb392. Likewise, staining of the *KIFB9^RNAi^* parasite revealed a discontinuous signal, as observed for several PFR proteins. Second, imunoblotting showed significant reduction in the amount of both PFR1 and PFR2 band intensities in *KIFB9^RNAi^* and *PFR2^RNAi^* mutants when compared to the WT. In imunoblotting of mutants, the recognition of upper (PFR1) protein band further confirms the targeting of PFR1 by Nb392 in a reduced condition. Notably, PFR2 band was recognized, indicating the presence of a residual PFR2 protein in *PFR2^RNAi^* mutants and also suggesting that Nb392 can recognize PFR2 only in reduced conditions corroborating PFR1/PFR2 similarity [37], [38].

The trypanosome flagellum contains up to 600 proteins [39]–[41]. Within the flagellum, the PFR is a complex, three-dimensional structure that runs alongside the microtubular axoneme [28], [42] and is vital for trypanosome motility [43] and survival *in vivo* [44]. The PFR alone contains more than 30 proteins [45], including PFR1 and PFR2 which are most abundant and encoded by a cluster of at least 5 identical genes each [36], [37]. The coding sequences of PFR1 is 67% identical to PFR2 [37]. Since more PFR are being discovered [41] highly specific, consistent and easy-to-produce markers are needed to optimize investigation into the PFR. Our results demonstrate that Nb392 is an excellent marker for the PFR. Furthermore, despite the PFR complexity, the successful isolation of PFR1 protein from an intrinsic protein mixture demonstrates the specificity of Nb392 for PFR protein. This will be useful in purification of this protein for vaccination, diagnosis and general research purposes. Furthermore, bloodstream and procyclic forms were used for flow cytometry and immunofluorescence experiments showing that Nb392 likely works on all parasite stages. Considering our immunization protocol, our result reiterates the immunodominance and abundance of PFR1 and PFR2 [46].

Finally, PFR proteins in *T. cruzi* were shown to be attractive targets for generating protective immunity against trypanosomes [47], [48]. Due to its immunogenic and abundant nature, as well as its high conservation in many parasitic species, PFR could be an ideal diagnostic target. Phylogenetic analysis of PFR sequences of kinetoplastids reveals closer relatedness among the genus *Trypanosoma* (*T. brucei, T. congolense* and *T. vivax*) while highlighting a distant relatedness to *T. cruzi* [49].

Combined, we demonstrate a novel immunoprecipitation capability of nanobodies by isolating PFR proteins from trypanosome lysate using Nb392. Being a recombinant antibody fragment, it can easily be adapted to various tracking or detection devices such as the lateral flow dipstick. It can also serve as an interesting alternative to already existing mouse anti-PFR monoclonal antibodies [38] for multiple staining by IFA if biotinylated or labelled with fluorophore. However, the negative ELISA signals obtained on *T. congolense* and *T. vivax* lysates limit the use of Nb392 as a species cross reactive antigen-ELISA antibody. Hence, work should be done to optimize the amplification of the signals or improve the avidity of Nb392 to these strains. As such, possibility of biotinylated or bivalent Nb392 constructs should be explored. Apart from lysates, serum antigen detection should be validated. Compared to conventional monoclonal antibodies, Nbs are heat stable, the development of Nb392 opens a new dimension and shows the potentials of the use of nanobody technology for diagnostic, crystallization and other protein research purposes in trypanosomes and other disease agents. With respect to the use of Nb392 for diagnosis of trypanosomiasis, the fact that Nb392 cannot distinguish between PFR proteins of different trypanosome species is less important in AAT treatment. This is because the treatment of AAT is done with similar drugs irrespective of the species responsible hence, the cross- reactivity of Nb392 may even be considered an advantage.

## Supporting Information

S1 Fig **Flagellum purification.** Sample of purified flagella from *T. evansi* STIB 816 viewed by bright-field microscopy. Magnification X1000.
(TIF)

S2 Fig **Mass spectrometric analysis of immunoprecipitated flagellar proteins.** Proteins were digested with trypsin and analysed on mass spectrometer. Upper (∼73 kDa) protein band (S2A), lower (∼69 kDa) protein band (S2B).
(TIF)

S1 Material and Methods Supporting material and methods including the Trypanosome antigen preparation, Flow cytometry and Immunofluorescence Assay (IFA), Western Blotting, Flagellum purification, Maldi-MS of membrane bands and supplementary reference.
(DOCX)
