# Supplementary figures and images for: Generation of a Nanobody Targeting the Paraflagellar Rod Protein of Trypanosomes
Source: PLoS One. 2014 Dec 31;9(12):e115893. doi: 10.1371/journal.pone.0115893 (PMC4281110; doi:10.1371/journal.pone.0115893)

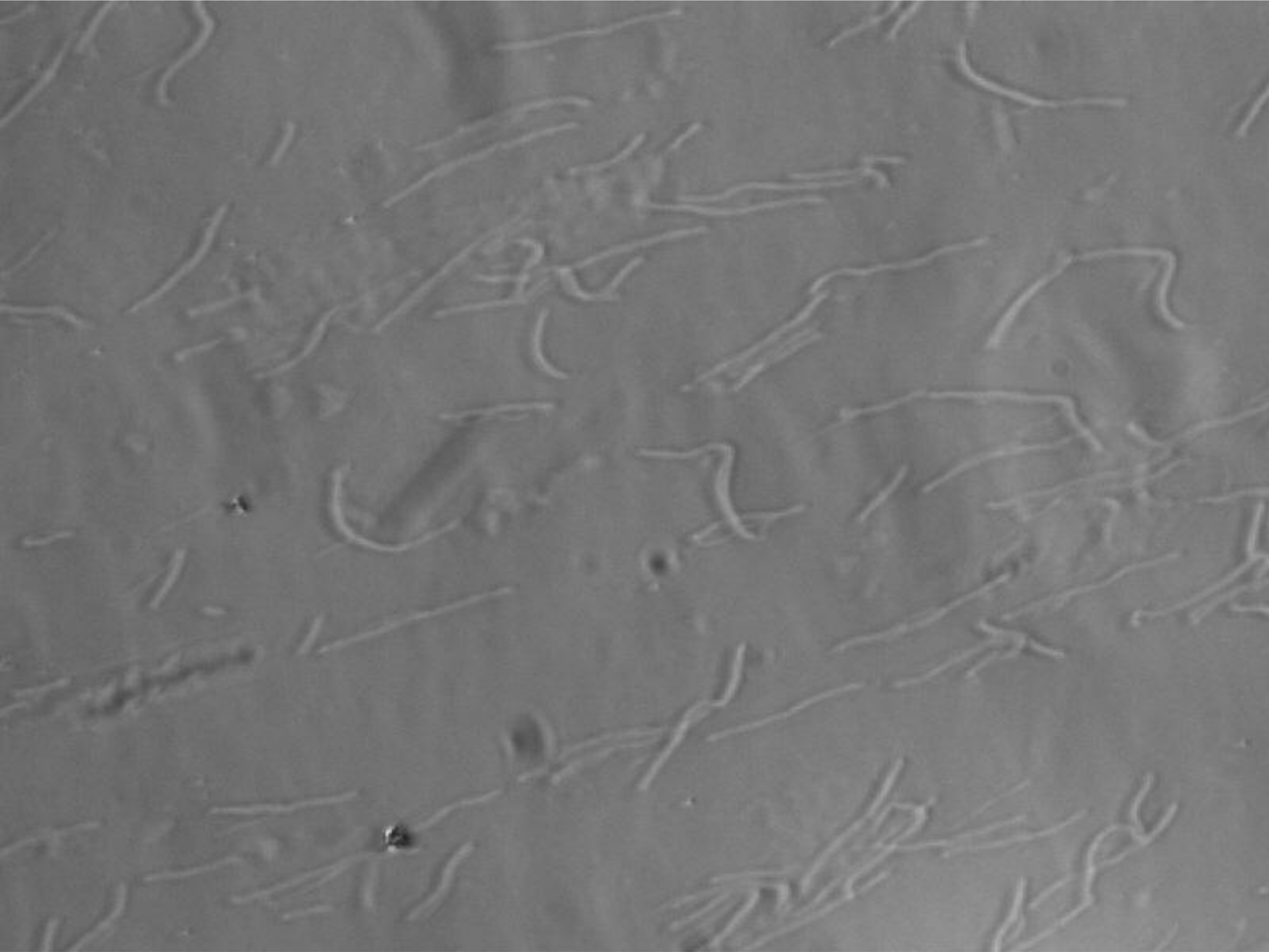

Supplement: S1 Fig — Flagellum purification. Sample of purified flagella from T. evansi STIB 816 viewed by bright-field microscopy. Magnification X1000. (TIF) [file pone.0115893.s001.tif]

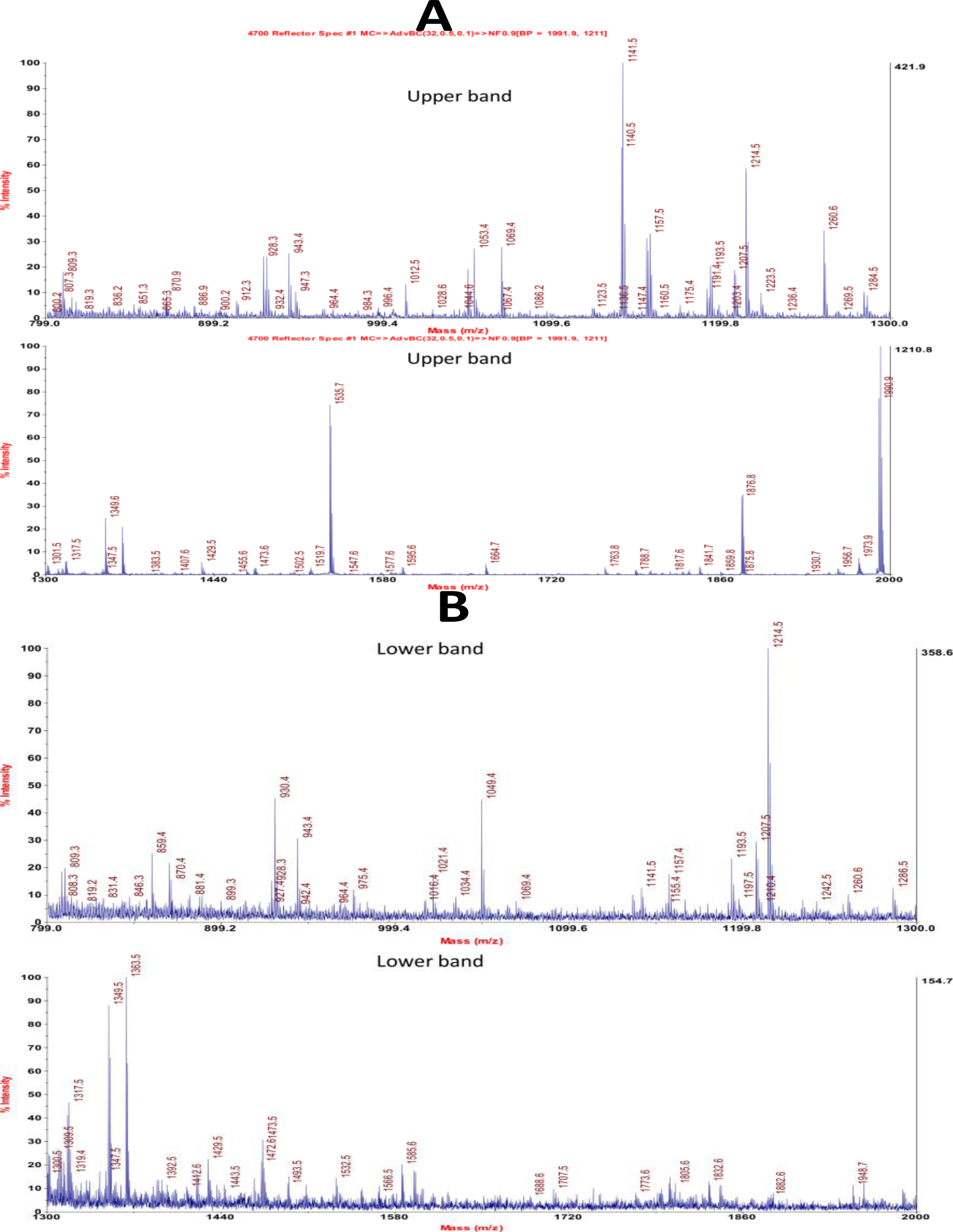

Supplement: S2 Fig — Mass spectrometric analysis of immunoprecipitated flagellar proteins. Proteins were digested with trypsin and analysed on mass spectrometer. Upper (∼73 kDa) protein band (S2A), lower (∼69 kDa) protein band (S2B). (TIF) [file pone.0115893.s002.tif]
