# Supplementary material for: Generation of a Nanobody Targeting the Paraflagellar Rod Protein of Trypanosomes
Source: PLoS One. 2014 Dec 31;9(12):e115893. doi: 10.1371/journal.pone.0115893 (PMC4281110; doi:10.1371/journal.pone.0115893)
Supplement: S1 Material and Methods — Supporting material and methods including the Trypanosome antigen preparation, Flow cytometry and Immunofluorescence Assay (IFA), Western Blotting, Flagellum purification, Maldi-MS of membrane bands and supplementary reference. (DOCX) [file pone.0115893.s003.docx]

**Supplemental information for**

**Generation of a nanobody targeting the Paraflagellar Rod protein of trypanosomes**

Emmanuel Obishakin^1, 2*^, Benoit Stijlemans^1, 3^, Julien Santi-Rocca^5^, Isabel Vandenberghe^4^, Bart Devreese^4^, Serge Muldermans^1, 2^, Philippe Bastin^5^, Stefan Magez^1, 2^.

*^1^ Cellular and Molecular Immunology, Vrije Universiteit Brussel, Pleinlaan 2, B-1050 Brussels, Belgium*

*^2^ Structural Biology Research Center, VIB (Flanders Institute for Biotechnology), Pleinlaan 2, B-1050 Brussels, Belgium*

*^3^Laboratory of Myeloid Cell Immunology, VIB, (Flanders Institute for Biotechnology) Pleinlaan 2, B-1050 Brussels, Belgium*

*^4^Laboratory for Protein Biochemistry and Biomolecular Engineering (L-ProBE), Department of Biochemistry and Microbiology, Ghent University, K.L Ledeganckstraat 35, 9000, Ghent, Belgium*

*^5^Trypanosome Cell Biology Unit, Institut Pasteur & CNRS, URA 2581, 25 rue du Docteur Roux, 75015 Paris, France.*

^*^Corresponding author : Emmanuel Obishakin, Cellular and Molecular Immunology, Vrije Universiteit Brussel, VIB, Pleinlaan 2, B-1050 Brussels, Belgium

(e-mail: eobishak@vub.ac.be). Phone: +3226291976, Fax: +3226291981

Abstract: none

**Supplemental Materials and Methods**

**Trypanosome antigen preparation**

10 week old female C57Bl/6 mice were infected subcutaneously with 5,000 parasites from stabilates of different *T. evansi* strains. On the sixth day, heparinised blood was collected and parasites were isolated from blood using DE52 as described [[1](#_ENREF_1)]. Parasites were counted on light microscope and washed four times in PBS by centrifugation to eliminate mouse serum proteins, pellet was resuspended at the rate of 10^8^ parasites per ml of cOmplete™ protease inhibitor (Roche) (1 tablet/50 ml PBS). After three rounds of freeze thawing at -80^0^C, lysates of trypanosomes were prepared by sonicating three times for 10 seconds each (Soniprep 150, SANYO^®^). The lysate was centrifuged at 14,000 rpm for 30 minutes, supernatant was collected and quantified using Nanodrop ® spectrophotometer ND-1000 (NanoDrop Technologies) and was stored at -20 ^0^C until further use.

**Flow cytometry and Immunofluorescence Assay (IFA)**

Parasites of each species were transferred into eppendorf tube in PSG solution and centrifuged at 800 x g for 10 minutes. The cell pellet was fixed and permeabilised by gently resuspending it in 100µl of BD Cytofix^TM^ for 20 minutes incubation on ice, washed with BD Perm/Wash ^TM^ washing buffer at 800 x g for 10 minutes. Cells were labeled by adding 1-1.5 µg of ALEXA conjugated nanobody to the permeabilised cells and incubated on ice for 30 minutes, washed with accompanying washing buffer at 800 x g for 10 minutes. 50 µl of 1/50 dilution of DAPI (4’, 6-diamidino-2-phenylindole) was added to the permeabilised cells. Flowcytometry analyses were performed on a FACS Canto II. The labeled cells were observed with Nikon ECLIPSE E600 microscope. Unlabeled parasites and ALEXA labelled Nb BCII 10 were used as negative control.

**Western Blotting**

For the initial Western blotting, 200 µg of parasite lysates prepared as above (Trypanosome antigen preparation) were used. After the addition of reducing agent to the samples (NuPAGE® Sample Reducing Agent (10X) and NuPAGE® LDS Sample Buffer (4X), the samples were boiled at 95ºC on a heating block. The samples were later run on a 12.5% SDS-PAGE and subsequently transferred onto a nitrocellulose or polyvinylidene difluoride (PVDF) membrane. Next, the membrane was blocked with 5% milk in PBS overnight at 4°C with gentle rocking. The membrane was washed with 0.05% Tween 20 in PBS for 5 minutes thrice in a Petri dish while gently rocking on a shaker, followed by incubation with 10ml of nanobody 392 at 5µg/ml in 1.5% non-fat dry milk (w/v) for 1 h at 4°C while gently rocking. After washing as above, the membrane was incubated in 10 ml of 1:1000 rabbit anti-VHH in 1.5% milk for 1hr at 4°C. After washing, it was followed with the incubation in 1:1000 of HRP-conjugated anti-rabbit IgG in 1.5% milk for 1 h at 4°C. Subsequently, the reaction was developed using 4-chloro-1-naphthol for chromogenic detection of horseradish peroxidase (HRP) activity. On the other hand, 10^6^ parasites were used for the Western blotting using both the wild type and RNAi mutant parasites. Enhanced chemiluminescence (ECL) method was employed to develop the Western blot in this case (classic ECL revelation (Amersham RPN 2106 "ECL Western Blotting Detection Reagents).
 Quantification was done with Image J software.

**Flagellum purification**

2.52 x 10^9^ DEAE 52-purified *T. evansi* STIB816 trypanosomes were washed four times with PSG at 800 x g for 10 minutes. The pellet was resuspended in 30ml of 0.1% Triton X-100 + MME buffer (10mM mops, pH 6.9, 1mM EGTA, 1mM MgSO_4_) for 10 minutes on ice, centrifuged twice at 3,000g for 10 minutes at 4^0^C to obtain cytoskeleton as pellets. The cytoskeleton was resuspended in 0.1% TritonX-100+1mMNacl+MME buffer for 10 minutes on ice. Flagella (pellet) were collected by centrifugation at 16,000g for 10 minutes. Extracts were confirmed on the Nikon ECLIPSE E600 microscope at 60x (oil immersion) and subsequently sonicated 3 times for 10 seconds each using a sonicator (Soniprep 150, SANYO^®^).

**Maldi-MS of membrane bands**

The excised gels were submerged with 150 µl of 200 mM NH_4_HCO_3_ in 50% Acetonitrile and incubated at 30°C for 20 minutes. The latter procedure was repeated a second time. The gels were then dried in a Savant SpeedVac^®^ concentrator ( model SC110-210) and cooled on ice prior to the addition of 10 µl of digest buffer, consisting of 50 mM NH_4_HCO_3_ , pH 8,5 and containing 0.02 µg modified trypsin (Promega, Madison, USA). Further, gel slices were incubated on ice for 45 minutes, diluted with 40 µl of digest buffer and incubated overnight at 37°C. Tryptic peptides were harvested by the separate collection of each supernatant, followed by a two-step extraction of the gel slices with respectively 40 and 20 µl of 60% acetonitrile in 0.1% formic acid at 30°C for 20 minutes, followed by 3 minutes strong shaking. The total volume, consisting of the original supernatants to which the extracts were added, were Speedvac dried on the SpeedVac^®^ concentrator and re-dissolved in 10 µl of 0.2% formic acid. The peptide extracts were spotted onto an Opti-TOF® 384 Well MALDI Plate Insert (ABSCIEX, Framingham, USA), in a 1/1 ratio mix with MALDI matrix α-cyano-4-hydroxy cinnamic acid (sigma,) prepared in a concentration of 5 mg/ml in 0.1% TFA/10 mM ammoniumcitrate/50% Acetronitrile. Sample Ms and tandem Ms spectra were acquired on a 4800 Proteomics Analyzer, a MALDI-TOF-TOF instrument (ABSCIEX, Framingham, USA) , using the delayed extraction and reflector technologies in the positive ion mode. Default settings were used that includes a bin size of 2 ns, a final detector voltage of 1840, and a delay extraction time of 430 ns. Laser intensities were set between 4200 and 4500 in MS mode and 5000 to 5500 in tandem Ms mode. Each spectrum was the sum of 1200 laser shots. The instrument was calibrated with Glu-Fibrinopeptide standards (*Calmix 4700* calibration standard, Applied Biosystems). The acquisition methods were factory methods. The MS/MS, 1 kV, CID off, positive method included a relative precursor mass window of 200 (FWHM) and a random pattern of shots.

**Supplementary reference**

1. Lanham SM, Godfrey D. Isolation of salivarian trypanosomes from man and other mammals using DEAE-cellulose. Experimental parasitology **1970**; 28:521-34.
